# Diagnosis of pathological conditions of kidney by two-dimensional and three-dimensional ultrasonographic imaging in dogs

**DOI:** 10.14202/vetworld.2016.693-698

**Published:** 2016-07-04

**Authors:** Dinesh Dehmiwal, S. M. Behl, Prem Singh, Rishi Tayal, Madan Pal, R. K. Chandolia

**Affiliations:** 1 Department of Veterinary Surgery and Radiology, Lala Lajpat Rai University of Veterinary and Animal Sciences, Hisar, Haryana, India; 2 Department of Veterinary Gynaecology and Obstetrics, Lala Lajpat Rai University of Veterinary and Animal Sciences, Hisar, Haryana, India

**Keywords:** hydronephrosis, hyperechoic, hypoechoic, nephritis, ultrasonography

## Abstract

**Aim:**

The objective of the study was to obtain and compare two-dimensional (2D) and three-dimensional (3D) ultrasonographic images of the kidney in different disease conditions.

**Materials and Methods:**

In this study, 11 clinical cases of different age groups of dogs suffering from kidney diseases were diagnosed by 2D and 3D ultrasonography at Teaching Veterinary Clinical Complex, Lala Lajpat Rai University of Veterinary and Animal Sciences, Hisar. The ultrasound (US) machine used for this study was 3D US machine (Nemio-XG: Toshiba, Japan) having four-dimensional (4D) volumetric probe. The images were acquired with 3-6 MHz 2D curvilinear transducer and 4.2-6 MHz 4D volumetric curvilinear transducer.

**Results:**

Nephritis was diagnosed in four dogs aged between 5 months and 6 years. In all the cases of nephritis diffuse increase in echogenicity of kidney, parenchyma was observed. Two dogs with end-stage kidney disease were also diagnosed. In both 2D and 3D ultrasonography, the kidney size was decreased and architectural details were also lost in them. The cases of regional renal diseases diagnosed were hydronephrosis and nephrolithiasis. Dilated renal pelvis was the common finding in all the three cases of hydronephrosis in both 2D and 3D ultrasonogram. Nephroliths were observed in one case with the history of hematuria and oliguria. The multifocal renal disease diagnosed in this study was dysplastic polycystic kidney. In 2D ultrasonogram, six anechoic cavities appeared with thin strip of renal parenchyma. In 3D ultrasonogram, the cysts appeared as black anechoic areas.

**Conclusion:**

The result of the current study showed that the clinical conditions of kidney such as nephritis, end-stage kidney, hydronephrosis, polycystic kidney, and nephrolithiasis can be diagnosed easily using 2D and 3D ultrasonography. Visualization of renal structures was clear in 2D ultrasonography in the conditions of nephritis and end-stage kidney. However, the conditions such as hydronephrosis, polycystic kidney, and nephrolithiasis were visualized clearly in both 2D and 3D ultrasonography equally.

## Introduction

Ultrasound (US) imaging is usually one of the first studies performed to assess the kidneys because important anatomic information concerning the size, shape and internal architecture can be obtained even in the presence of impaired renal function or abdominal fluid [1].

The US is furthermore useful for guiding interventional procedures [2]. The three-dimensional (3D) ultrasonography, available in medicine for over 10 years enabled great advances in the area of diagnostic imaging. This technique facilitates the volumetric study of organs and structures, besides it, provide a third image plane, the coronal plane which allows more precisely volumetric calculation mainly those of irregular shapes.

The 3D ultrasonography is still a new technique in veterinary medicine, and few are the scientific papers that report its experimental use [3]. The objective of the study was to obtain and compare two-dimensional (2D) and three-dimensional (3D) ultrasonographic images of the kidney in different disease conditions.

## Materials and Methods

### Ethical approval

The study was conducted after the approval of Institutional Animal Ethics Committee.

### Study site

The study was conducted in the Department of Veterinary Surgery and Radiology with the collaboration of Department of Veterinary Gynaecology and Obstetrics, College of Veterinary Sciences, Lala Lajpat Rai University and Animal Sciences (LUVAS), Hisar (Haryana).

### Animals

The dogs (n=11) with pathological conditions of kidneys reported to Teaching Veterinary Clinical Complex (TVCC), College of Veterinary Sciences, LUVAS, Hisar (Haryana) were used for the study.

### Ultrasonographic examination

The study was conducted on 11 clinical cases of different age groups of dogs suffering from kidney diseases brought to TVCC, LUVAS, Hisar. The dogs were sedated with xylazine at 1 mg/kg body weight for restraining. For scanning of kidneys, the area caudal to the 11^th^ rib up to the pubis on either side of the midline, up to the level of transverse process of lumbar vertebrae was shaved properly. The dogs were controlled in lateral recumbency, i.e., left lateral recumbency for right kidney and right lateral recumbency for left kidney. The left kidney was scanned below the transverse process of first to third lumbar vertebrae and for the right kidney, in addition, an intercostal approach through the last or second last intercostal space was used to scan it in its transverse plane. Scanning area was shaved properly and enough gel was applied over the site and the surface of the transducer to get a better image. The US machine used for this study was 3D US machine (Nemio-XG: Toshiba, Japan) having four-dimensional (4D) volumetric probe. The images were acquired with 3-6 MHz two-dimensional (2D) curvilinear transducer and 4.2-6 MHz 4D volumetric curvilinear transducer.

## Results

The various conditions of kidney diagnosed were nephritis (n=4), end-stage kidney (n=2), hydronephrosis (n=3), polycystic kidney (n=1), and nephrolithiasis (n=1).

### Nephritis

**Figure-1:**
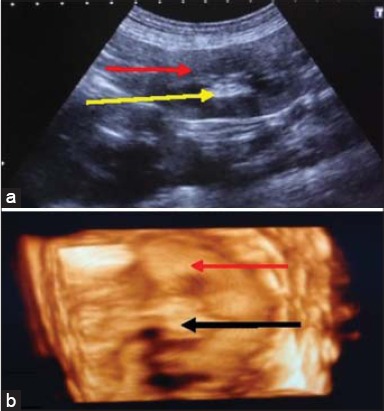
Nephritis (Case 1): (a) In two-dimensional ultrasonogram of kidney, the echogenicity of renal parenchyma has increased without differentiation of cortex and medulla (red arrow). Echogenic renal crest (yellow arrow), (b) in three-dimensional (3D) renal parenchyma without distinction of cortex and medulla (red arrow). Renal crest visible in the center (black arrow).

**Figure-2:**
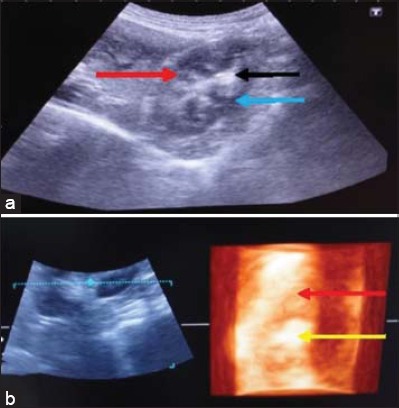
Nephritis (Case 4): (a) In two-dimensional ultrasonogram of kidney, the echogenic of renal diverticulae (red arrow), renal crest (black arrow) and corticomedullary junction (blue), (b) in three-dimensional ultrasonogram of kidney, homogeneous without differentiation of medulla (red arrow). Echogenic renal sinus in the center (yellow arrow).

Nephritis was diagnosed in four dogs aged between 5 months and 6 years. In the first case, a dog was brought to TVCC with the history of anorexia since 20 days, epistaxis, scanty urine with increased values of blood urea nitrogen (BUN) and creatinine. In 2D ultrasonogram (Figure-1a), there was a lack of distinction between cortex and medulla. The echogenicity of cortex and medulla was found to be increased. Renal margins were also not clearly demarcated. In 3D ultrasonogram (Figure-1b), the renal parenchyma appeared as a continuous structure without the distinction of cortex and medulla. The margins of kidney were more clearly demarcated in 3D. In the second case, a bitch was brought to TVCC with the history of continuous vomition, anorexia and not responding to the treatment. The 2D ultrasonogram revealed homogenous renal parenchyma without demarcation of cortex and medulla and increased echogenicity. The renal sinus appeared as a bright echogenic area. In 3D ultrasonogram, the renal parenchyma and renal margins were clearly visualized. In both 2D and 3D ultrasonography, the renal cortex and medulla were not distinct. In the third case, a pup of 6 months age with the history of anorexia, intermittent vomition and stunted growth was brought to TVCC. In 2D ultrasonogram, the kidney appeared as a hyperechoic structure without demarcation of cortex and medulla. Renal margins were clearly visualized. In 3D ultrasonogram, the renal parenchyma was more clearly visualized. The other renal structures were poorly visualized. In the fourth case, a dog was brought to the TVCC with the history of anorexia, vomition and hematuria since 5 days. In 2D ultrasonogram (Figure-2a), the renal architecture was clearly visualized. The echogenic renal sinus and renal diverticulae were clearly visible. In 2D ultrasonogram, renomegaly was observed and the corticomedullary junction was also clearly demarcated. In 3D ultrasonogram (Figure-2b), the renal parenchyma was visualized without clear demarcation of cortex and medulla. The echogenic renal sinus was visualized clearly. Renal margins were not clearly demarcated.

### End-stage kidney

**Figure-3:**
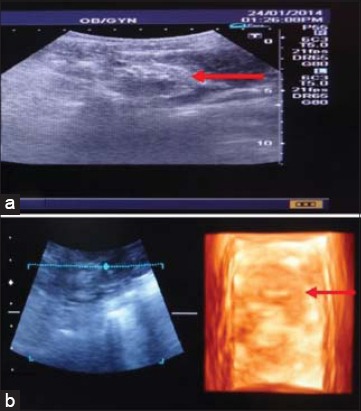
End-stage kidney (Case 1): (a) In two-dimensional ultrasonogram of kidney, small sized kidney appears without distinct margins (red arrow) and differentiation of cortex and medulla, (b) in three-dimensional ultrasonogram of kidney, small sized kidney appears without distinct margins (red arrow).

In this study, two cases of end-stage kidney were diagnosed with the help of ultrasonography. In the first case, a cocker spaniel dog of age 7 years was brought to the TVCC with the history of anorexia and vomition since 5 days. Furthermore, there was blood in the vomitus. The values of BUN and creatinine were 295 and 12.5 mg/dl, respectively. In 2D ultrasonogram (Figure-3a), the margins of kidney were not clear. The renal architecture was poorly visualized with no distinction of cortex and medulla. The renal parenchyma appeared as an area of mixed echogenicity with both hypoechoic and hyperechoic areas. Renal sinus was the most distinct structure in the whole kidney. In 3D ultrasonogram (Figure-3b) also the kidney architecture was poorly visualized. Kidney margins were also not clear. The renal sinus appeared as an echogenic area. Kidney parenchyma was visualized without distinction of cortex and medulla. In the second case, a dog of 13 years age was brought to the TVCC with the history of anorexia, continuous vomition since 6 days and urine incontinence since 1 week. The values of BUN and serum creatinine were 161 and 16 mg/dl, respectively. In both 2D and 3D ultrasonogram, there was no demarcation of kidney margins. The renal architecture was poorly visualized with no distinction of renal structures. The renal parenchyma appeared as a structure of mixed echogenicity. It was more clearly visualized in 3D ultrasonogram.

### Hydronephrosis

**Figure-4:**
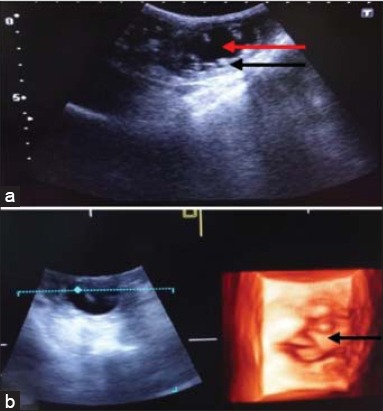
Hydronephrosis Case 1: (a) In two-dimensional ultrasonogram of kidney with dilated renal pelvis (red arrow). Atrophied renal parenchyma with loss of architectural details (black arrow), (b) in three-dimensional ultrasonogram dilated renal pelvis (red arrow).

**Figure-5:**
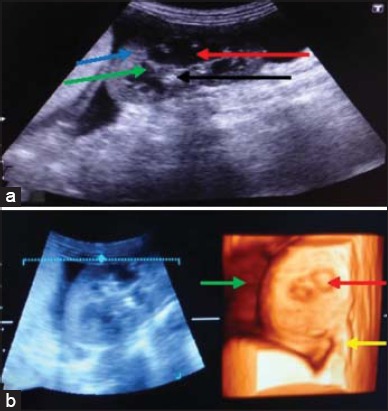
Hydronephrosis Case 3: (a) In two-dimensional ultrasonogram of kidney with dilated renal pelvis (red arrow). Atrophied renal parenchyma with loss of architectural details (black arrow), (b) in three-dimensional ultrasonogram fluid filled areas (red arrow), course of ureter (yellow arrow) and ascetic fluid outsite the kidney (green arrow).

In this study, hydronephrosis was observed in two cases of urinary tract obstruction and one case of ascites. In the first case, a dog was brought to the TVCC with the history of constipation, urine incontinence, and anorexia since 8 days. The values of BUN and serum creatinine were 326 and 13.3 mg/dl, respectively. In 2D ultrasonogram (Figure-4a), dilated anechoic renal pelvis was observed. Due to obstruction back pressure of urine causes atrophy of renal parenchyma. The renal cortex appeared thinner. There was acoustic enhancement distal to the dilated renal pelvis. The corticomedullary junction and interlobar vessels were not distinct. In 3D ultrasonogram (Figure-4b), the dilated renal pelvis was visualized clearly. The renal pyramids were also visualized. There was no distinction of cortex and medulla. The margins of kidney were also not clearly demarcated. In the second case, a dog of 6 year age was brought to the TVCC with the history of urine incontinence since 10 days. The values of BUN and creatinine were 142 and 18 mg/dl, respectively. The urinary bladder of dog was palpable from body wall. In 2D ultrasonogram, renal pelvis appeared dilated with anechoic lumen. Poor corticomedullary junction distinction and mixed echogenicity of renal parenchyma were observed. Kidney margins were clearly demarcated. In 3D ultrasonogram, the visualization of renal architecture was more clear. The dilated renal pelvis with anechoic lumen was clearly visualized. Kidney margins were also clearly visualized. In the third case, a Rottweller dog of 16 months age was brought to the TVCC with the history of anorexia since 2 months and blood in vomitus and urine. Body condition was very poor. The abdomen of dog was distended with the fluid of ascites. In 2D ultrasonogram (Figure-5a), all the visceral organs were clearly visualized due to fluid in the abdominal cavity. The kidney architecture was clearly visualized. The renal parenchyma appeared as a homogenous structure without distinction of cortex and medulla. Three focal hypoechoic areas were visualized in the medullary regions. Renal sinus was hyperechoic. The renal pelvis was not visualized. Hydronephrosis appeared due to fluid in the renal parenchyma and not due to ureteral obstruction. In 3D ultrasonogram (Figure-5b), focal hypoechoic areas appeared in the renal parenchyma.

The renal architecture and renal margins were clearly demarcated. The cortex and medulla appeared distinct.

### Nephrolithiasis

**Figure-6:**
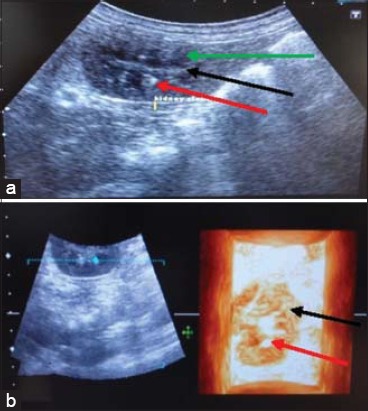
Nephrolithiasis: (a) In two-dimensional ultrasonogram of kidney the nephrolith is clearly visible (red arrow). The architectural details showing renal crest (black arrow) and divarticulae (green arrow), (b) in three-dimensional ultrasonogram of kidney the nephrolith (red arrow) and renal parenchyma (black arrow).

Nephroliths were observed in one case with the history of hematuria and oliguria. A Pomeranian dog of age 12 years was brought to the TVCC with the history of oliguria and blood mixed urine since 7 days. A radiograph revealed the cystoliths in the urinary bladder but no information about nephroliths was obtained. In 2D ultrasonogram (Figure-6a), the renal cortex and medulla were more echogenic than normal and corticomedullary junction was also poorly visualized. A small hyperechoic shadow of nephrolith near the diverticula was clearly visible. Renal diverticulae and kidney margins were clearly visualized.

In 3D ultrasonogram (Figure-6b), the renal architecture was more clearly visualized. The renal sinus and diverticulae were also more clear than 2D ultrasonogram. The nephroliths appeared as a small hyperechoic structure in the renal parenchyma.

### Polycystic kidney

**Figure-7:**
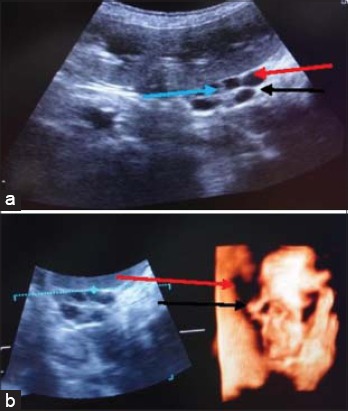
Polycystic kidney: (a) In two-dimensional ultrasonogram of kidney, six anechoic fluid filled structures (red arrow), thin renal parenchyma with indistinct kidney margins (black arrow) and renal divarticulae (blue arrow), (b) in three-dimensional ultrasonogram of kidney, anechoic fluid filled structures (red arrow), and renal diverticulae (black arrow).

The multifocal renal disease diagnosed in this study was dysplastic polycystic kidney. A black Labrador dog of age 6 years was brought to the TVCC with the history of scanty feces, anorexia, scanty urination, and epistaxis. The BUN and creatinine values were 282.0 and 9.3 mg/dl, respectively. In 2D ultrasonogram (Figure-7a), six anechoic fluid filled structures were observed in the renal parenchyma. The cortex appeared thinner than normal. The margins of kidney were also not clearly seen. The acoustic enhancement distal to these fluid filled structures was also clearly visualized. Renal sinus and diverticulae were clearly visible. In 3D ultrasonogram (Figure-7b), the anechoic lumen of cysts was visualized but there boundary was not distinct. The renal sinus and diverticulae were more prominent than 2D ultrasonogram.

## Discussion

US is the most commonly used imaging method for studying urinary tract disorders in dogs, as it is easy to perform, inexpensive and provides excellent contrast resolution in real-time [4]. A large number of studies have been carried out to access and enumerate sonographic features of the kidney, ureter, urinary bladder, and urethra both in healthy and diseased dogs. However, sonographic features of any urinary disease are not specific. Therefore, clinical and laboratory findings are required along with ultrasonography to arrive at a definitive diagnosis [5]. Diseases of the kidney diagnosed by ultrasonography can be divided into diffuse renal diseases, regional renal diseases, and focal or multifocal renal diseases. The diffuse renal diseases diagnosed were nephritis and end-stage kidney. The regional renal diseases diagnosed were hydronephrosis and nephrolithiasis. The multifocal renal disease diagnosed was polycystic kidney [1].

Diffuse renal diseases observed in dogs are mainly glomerulonephritis, chronic interstitial nephritis, and end-stage kidney. They are most difficult to evaluate ultrasonographically as compared to regional and multifocal renal diseases and creates a diffuse increase in echogenicity of kidney parenchyma [6]. Verma *et al*. [5] also observed hyperechoic cortex and medulla in the case of nephritis. The ultrasonographic features are not specific to further differentiate between glomerulonephritis and interstitial nephritis. According to Finn-Bodner [6], the major ultrasonographic findings of glomerulonephritis are hyperechoic medulla and cortex or diffuse increase in renal echogenicity. In interstitial nephritis, there is infiltration of inflammatory cells and exudates as well as the proliferation of fibrous tissue [7]. These results in increased echogenicity of renal parenchyma. Thus, the ultrasonographic features are not specific and require renal biopsy, hematological, biochemical, and urological observations for confirmatory diagnosis. Nephritis was diagnosed in four dogs aged between 5 months and 6 years. In all the cases, partial or full anorexia, intermittent vomition, dehydration, and lethargy were common signs. In two cases, the values of BUN and creatinine were very high. Hematuria was present in one case. In one of the case, there was a pup of 5 months age with partial anorexia, intermittent vomition, stunted growth, weakening of limbs and rough hair coat. Ultrasonography revealed increased echogenicity of renal parenchyma. This suggested the possibility of interstitial nephritis in the pup. The interstitial nephritis is associated with leptospirosis and infectious canine hepatitis in dogs [7]. The pup was vaccinated and the disease may be occurred due to vaccination failure.

End-stage kidney was the other diffuse renal disease diagnosed by ultrasonography in two dogs of age 13 and 7 years respectively. The values of BUN and creatinine were found to be increased in both the cases. Prolonged anorexia, persistent vomition, and oliguria were common clinical signs. One of the dogs was suffering from prostate enlargement and the other one was suffering from cystitis. The hemogram of dogs revealed anemia and neutrophilia. Erythropoietin deficiency may be the main cause of anemia, while azotemia may be the result of the loss of renal function and reduction in glomerular filtration rate [8]. In 2D ultrasonogram, margins of the kidney were not clear and decrease in the size of the kidney was observed. Finco *et al*. [9] observed that the decreased kidney size is related with chronic renal failure. The architectural details were also lost in both the cases. Similar findings were reported earlier by Verma *et al*. [5]. According to Felkai *et al*. [10], the loss of architectural details is a significant feature of chronic renal disease and occurs due to gradual loss of nephrons over a period of time. Thus, the ultrasonographic features along with clinical and laboratory findings were suggestive of end-stage kidney.

Regional renal diseases affect an anatomic or functional compartment of the kidney and are easier than diffuse renal diseases to diagnose by ultrasonography. They include pyelonephritis, hydronephrosis, nephrocalcinosis, nephrolithiasis, and hypercalcemic nephropathy. Urinary tract infection is common in dogs; especially in females is most often happening the result of ascending bacterial contamination of vulva, perivascular skin and vestibule [11]. In this study, the cases of regional renal diseases diagnosed were hydronephrosis (n=3) and nephrolithiasis (n=1). In both hydronephrosis and pyelonephritis renal pelvis dilatation occurs. According to Ackerman [12], Feeney and Walter [13] and Lamb [14] the hydronephrosis can create cyst-like expansion of the kidney. Pyelonephritis is unlikely to cause this extreme amount of pelvic dilatation. Thus, the differentiation of hydronephrosis and pyelonephritis is difficult with ultrasonography. According to Sastry and Rama Rao [7], hydronephrosis is the result of complete or partial obstruction of the urine flow from the affected kidney. Nyland *et al*. [1] reported that the renal pelvis should not be identifiable sonographically in normal dogs and cats. The urinary calculi, hemorrhagic cystitis, prostate enlargement, neoplasms and gravid uterus can cause urine obstruction and development of hydronephrosis. In this study, two cases of urinary tract obstruction and one case of ascites were observed with hydronephrosis. In 2D ultrasonogram, dilated renal pelvis was the common finding in all the three cases. In one case of urine obstruction the hydronephrosis was severe and kidney parenchyma becomes atrophied. In another case increased medullary echogenicity, poor differentiation between cortex and medulla and dilated renal pelvis were observed. Similar findings were observed by Verma *et al*. [5]. In 3D ultrasonogram, the dilated renal pelvis and renal diverticulae were more clearly visualized.

Nephroliths were observed in one case with the history of hematuria and oliguria. Radiograph revealed the cystoliths in the urinary bladder but no information about nephroliths was obtained. Ultrasonography is valuable for precise localization of radiographically visible mineral opacities in the region of the kidneys and ureters [15]. Nephroliths usually appear as intensely hyperechoic, discrete structures within the renal pelvis and collecting ducts [1]. Cystic calculi were also present in that case. In 2D ultrasonogram, the calculi appeared as a small hyperechoic structure near the caudal renal diverticula. No acoustic shadowing was observed. These findings were in agreement with the observations of Finn-Bodner [6] who opined that nephroliths may or may not create acoustic shadow. In 3D ultrasonogram, the nephrolith appeared as bright hyperechoic structure without changing their position.

The multifocal renal disease diagnosed in this study was dysplastic polycystic kidney. A black Labrador dog of age 6 years was brought to the TVCC with the history of scanty feces, anorexia, scanty urination and epistaxis. The 2D ultrasonogram revealed nephritis in left kidney and numerous cysts in right kidney. Six anechoic cavities appeared with thin strip of renal parenchyma. In 3D ultrasonogram, the cysts appeared as black anechoic areas. Konde [16] also described the renal cysts as circular anechoic structures with well-defined walls.

## Conclusion

The result of the current study showed that the clinical conditions of kidney such as nephritis, end-stage kidney, hydronephrosis, polycystic kidney, and nephrolithiasis can be diagnosed easily using 2D and 3D ultrasonography. Visualization of renal structures was more clear in 2D as compared to 3D ultrasonography in the conditions of nephritis and end-stage kidney. However, the visualization of renal structures in conditions such as hydronephrosis, polycystic kidney and nephrolithiasis was clear in both 2D and 3D ultrasonography equally.

## Authors’ Contributions

DD, SMB, and PS have designed the study and planned the research experiment. DD performed the research experiments. PS, RT, and RKC supervised the research. MP helps in conducting experiments. All the authors read and approved the final manuscript.
